# Nurse-Led Electrical External Cardioversion of Patients with Atrial Arrhythmia: A Systematic Review Update and Meta-Analysis

**DOI:** 10.3390/nursrep15020032

**Published:** 2025-01-23

**Authors:** Dalia Caleffi, Luca Pingani, Sergio Rovesti, Domenico Cannizzaro, Paola Ferri

**Affiliations:** 1 Clinical and Experimental Medicine PhD Program, Department of Biomedical, Metabolic and Neural Sciences, University of Modena and Reggio Emilia, Via G. Campi 287, 41125 Modena, Italy; domenico.cannizzaro@unimore.it; 2 Department of Biomedical Metabolic and Neural Sciences, University of Modena and Reggio Emilia, Via G. Campi 287, 41125 Modena, Italy; luca.pingani@unimore.it (L.P.); sergio.rovesti@unimore.it (S.R.); paola.ferri@unimore.it (P.F.)

**Keywords:** atrial fibrillation, external cardioversion, advanced nursing practice

## Abstract

**Background**: Atrial fibrillation, the most frequent and prevalent cardiac arrhythmia, often requires external cardioversion to ensure rhythm control. As healthcare professionals, nurses play a key role in autonomous intervention implementation. The aim was to update current evidence on the efficacy of nurse-led external direct current cardioversion. **Methods**: A systematic review of primary quantitative studies in English or Italian was conducted with no temporal filter. Seven database searches were interrogated. A total of nine articles were included, for which validity was evaluated and analysed. The review was performed using PRISMA guidelines for systematic reviews. Study characteristics were examined to determine if a meta-analysis was possible, and odds ratio was used as the effect size measure. **Results**: Data analysis led to the development of selected topics. The success rate of nurse-led direct current cardioversion appears to be high, at >80% (eight out of nine studies) in safe conditions. High-level professional training was required of nurses. There seemed to be no clear consensus on the management of anaesthetic aspects and medical support during the procedure. Meta-analyses of three studies found that there was no risk (M1-OR 0.89, CI [0.58, 1.36]; M2-OR 0.90, CI [0.59, 1.37]) difference between nurse-led DCCV and that performed by other clinicians. Few studies reported data on patient satisfaction, cost effectiveness, and waiting time. **Conclusions**: This review confirms that nurse-led external direct current cardioversion appears to be successful and safe in restoring sinus rhythm. A high level of nurse training and definition of a shared protocol could allow for effective implementation in more countries and settings.

## 1. Introduction

Atrial fibrillation (AF) is a cardiac arrhythmia with a prevalence among the adult population of >3% [1]. A worldwide increase in prevalence and incidence has been observed [2]. It is the most common cardiac arrhythmia [3], with a broad impact on all health services across primary and secondary care described by European Society of Cardiology (ESC) 2024 guidelines [4]. Most hospital admissions occur in patients ≥65 years of age. Patients >80 years of age are also associated with elevated in-hospital mortality [3]. AF is in fact correlated with a number of predisposing comorbidities such as hypertension heart disease, coronary disease, valvular heart disease, heart failure, hypertrophic cardiomyopathy, congenital heart disease, venous thromboembolic disease, obstructive sleep apnoea, obesity, diabetes, metabolic syndrome, and chronic kidney disease [5]. Stroke, ischaemic transient attack, systemic extracerebral embolism, cognitive impairment such as dementia, heart failure, and myocardial infarction are potential parallel complications that patients can develop if this arrhythmia is not adequately treated or identified [6].

Pharmacological cardioversion, external cardioversion or internal cardioversion with catheter ablation are three approaches used in the management of AF to obtain rhythm control and to restore the patient’s rhythm to normal (i.e., sinus rhythm) [4,7]. For a first AF episode, electrical cardioversion is preferred in most cases to avoid drug side effects, to avoid need for prolonged telemetric monitoring to screen for a proarrhythmic response and to reduce the risk of some antiarrhythmics to convert AF to atrial flutter [8]. AF catheter ablation is indicated for paroxysmal and persistent AF [4].

A multidisciplinary approach is considered fundamental in the management of patients with atrial fibrillation [4]. AF is a supraventricular arrhythmia, as are atrial flutter and atrial tachycardia [9]. Also, the most common procedure required to restore sinus rhythm for each of these arrhythmia conditions may be the same.

Restoring sinus rhythm or obtaining rate control are important goals of AF management. AF leads to the failure of normal functioning of the atrial chambers with the risk of complications and, in the long term, the development of heart failure and ischaemia [10].

Electrical cardioversion can be performed safely under short sedation and is effective in terminating AF in over 90% of cases [11]. The high prevalence of these arrhythmias is correlated with high necessity of cardioversion service; the necessity of effective patient care contributes to the development of advanced competencies.

Direct current cardioversion (DCCV) is one of the possible treatments to consider in AF rate and rhythm control [4]. AF treatment, which is associated with potential complications, is normally clinician-delivered.

However, as a non-invasive procedure with the development of advanced competencies, it may be possible to consider the involvement of other professionals. Nurses have the possibility to develop advanced competencies and be recognised as advanced practice nurses. They could increase knowledge, complex decision-making skills, and clinical competencies for expanded nursing practice and provide direct healthcare services [12].

Developing advanced competencies and involving other specialists in the independent management of non-emergent external cardioversion may help to redirect medical professionals to other important interventions such as AF ablation. Studies have demonstrated that reducing AF time to rhythm control, which includes cardioversion but also ablation, is important in improving outcomes and reducing postprocedural complications [13,14].

Two systematic reviews [15,16] analysed how an external direct current cardioversion could be implemented by a trained nurse specialist. However, the studies included in the systematic reviews were different in their methodologies, setting, training, and anaesthetic aspects of DCCV implementation. Considering the importance of the topic, our aim is to update the current evidence and to conduct the first meta-analysis on the efficacy of nurse-led elective and non-emergent external direct current cardioversion and correlated implications of its implementation. This could help to add evidence and to deepen knowledge about unclear aspects of the previous systematic review.

## 2. Materials and Methods

A systematic review was carried out through the guide of the Cochrane Library Handbook [17]. The report of the results followed the preferred reporting items for systematic review and meta-analysis (PRISMA) statement criteria [18]. The review protocol has been registered in Open Science Framework doi https://doi.org/10.17605/OSF.IO/2NBCR (accessed on 8 December 2024), and an article of the protocol with search strategies was published [19]. The PICO of the included studies summarises the question “What is the impact of a nurse-led external DCCV in AF on patient outcomes?”. The acronyms represent the following: (P) is patients with AF, (I) is nurse-led external elective cardioversion, (C) is usual practice, and (O) is health care outcomes. A secondary outcome investigated was the competence and training required of nurses. Usual practice meant conducting cardioversion as delivered in the study’s country. With the term clinicians, we mean a physician who is able to deliver this procedure in collaboration with physician assistants or other healthcare professionals.

This review included all studies conducted in a clinical setting where patients affected by atrial arrhythmia with an elective regime underwent a non-emergent electrical external cardioversion conducted by an expert or specialist nurse. Electrical external cardioversion conducted in different settings, such as an emergency context, for other arrhythmia types (i.e., ventricular tachycardia or ventricular fibrillation) or by other health care professionals (i.e., a physician or physician assistant) were excluded.

Secondary literature studies (e.g., reviews, meta-analyses, and study synopsis), descriptive articles that do not report data collected on a sample of subjects, case reports, case series, and expert opinions were not included.

The search strategy was developed with the support of an expert informatician. It was applied to many sources: Cinahl, Psycinfo, Embase, Scopus, Cochrane Library, PubMed, and Web of Science. Search strategies for each database were specified in the protocol (see Supplementary Materials Table S1). All articles, in the English or Italian language, with no temporal filter corresponding to inclusion criteria were selected.

After performing the searches, duplicate records were eliminated and screened by title and abstract by two independent reviewers. Any disagreement was actively discussed with the collaboration of a third researcher in case of discordance. To minimise the risk of bias, Rayyan software was used to ensure the process was blind [20]. With the same procedure, the full text of each potentially eligible study was analysed. A risk of bias tool (Rob 2) for randomised controlled trials [17] and Robins-I tool for non-randomised studies [21] were considered to assess the risk of bias. Grade approach with the development of a summary of findings was utilised to rate the certainty of evidence [22].

A critical assessment of the included studies was conducted by the two researchers using the risk of bias Cochrane tool. A data extraction format was compiled by two reviewers in order to have an overview of patients’ characteristics, settings, number of cardioversions, type of intervention, nurse training, complications, medical presence, anaesthetic support delivered, principal finding, data collection, and analysis.

We provided a narrative synthesis of the findings from the included studies. A synthesis of available data through meta-analysis was conducted to provide a consolidated estimate of the efficacy of nurse-led versus clinician DCCV. A fixed-effect method was utilised for the meta-analysis of the dichotomous variable; odds ratio was the effect measure calculated. The Higgins heterogeneity index and the I2 coefficient were calculated to determine the presence of heterogeneity. In the case of heterogeneity detection, the development of a stratified meta-analysis to cause determination was evaluated. Sensitivity analysis elaboration was considered in the analysis process to assess the robustness of the synthesised results [23].

## 3. Results

### 3.1. Selection of Sources Evidence

**Figure 1:**
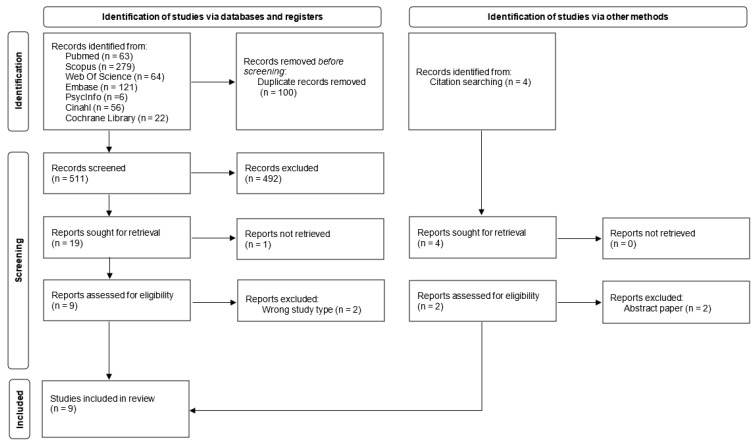
Flow diagram for selection of studies (PRISMA FLOW DIAGRAM).

Database-searching identified 611 articles; after removing duplicates, 511 articles were screened for title and abstract. Out of these articles, 19 were included for full-text review. Following a full-text review, seven articles were included. Another four potentially eligible articles were identified from full-text references analyses, of which two were excluded. A total of nine articles are included in this review. An outline of the study screening and selection process is presented in the PRISMA flow chart shown in Figure 1.

Two articles were considered eligible from the search strategies but were excluded after full-text analysis because the article types did not satisfy the inclusion criteria [24,25].

Critical appraisal of the studies included, conducted with the support of the Robins I tool for non-randomised studies for DCCV safety and effectiveness outcome, was carried out. See Supplementary Table S2 for a summary presented graphically using robvis [26] (green represents low risk, amber represents unclear risk, and red represents a high risk of bias). Confounding variables were not completely analysed or identified by all studies, and adjusted analysis was rarely conducted [27,28,29].

### 3.2. Characteristics of Source of Evidence

The included studies consist of three prospective studies (Boodhoo et al., 2004; Moore et al., 2014; Strzelczyk et al., 2017) [30,31,32], five retrospective studies (House et al., 2016; Norton et al., 2016; Purkayastha et al., 2023; Shelton et al., 2006; Zaher et al., 2023) [27,28,29,33,34] and one observational study (Quinn, 1998) [35]. Three studies were conducted in the USA [27,32,33], four in the United Kingdom [28,29,30,35] and one in Belgium [34]. The study participants were patients with atrial arrhythmia who received DCCV in a non-emergency context. The studies included cardioversion of inpatients and outpatients carried out in a monitored environment. Quinn (1998) [35] delivered cardioversion in a coronary care unit after outpatient assessment. A cardiac catheterisation laboratory was used by Boodhoo et al. (2004) [30] for all elective patients that were haemodynamically stable. Moore et al. (2014) [31] and Strzelczyk et al. (2017) [32] consider the cardioversion of outpatients and inpatients: the former in a high-dependency unit, and the latter in an electrophysiology laboratory. In the studies of Shelton et al. (2006) [29] and Zaher et al. (2023) [34], a cardiology day unit was utilised. Zaher et al. (2023) [34] specified that the cardioversions analysed were delivered after outpatient assessment. Two studies considered the outpatient care unit [27,32]. A generic monitored environment was the study setting of Purkayastha et al. (2023) [28].

### 3.3. Outcomes

The aim of all included studies was to focus on nurse-led or advanced practice provider (APP) atrial arrhythmias cardioversion. All studies analysed cardioversion efficacy in restoring sinus rhythm safety (Table 1) as well as any complication onset.

**Figure 2:**
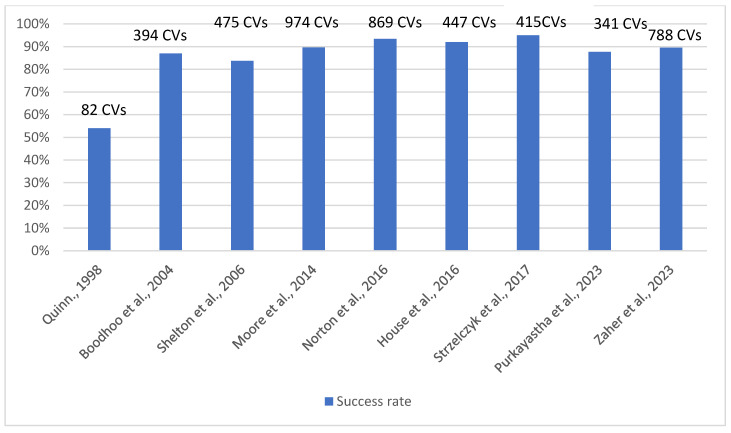
Success Rate [27,28,29,30,31,32,34,35].

As represented in Figure 2, Quinn (1998) [35] reports a success rate of 54% on a sample of 82 cardioversions performed. Adverse events such as major convulsion, moderate heart failure, and bronchospasm were observed in three patients. Boodhoo et al. (2004) [30], with 394 cardioversions performed on 300 patients, discovered a success rate of 87%, with 48% of patients in sinus rhythm at six weeks after the procedure. No immediate complications were reported, but four patients experienced systemic emboli up to four days after the procedure despite adequate anticoagulation. Minor symptoms of dermal injury at the sites of paddle application were reported by 40% of patients who responded to the tolerability questionnaire. Shelton et al. (2006) [29] analysed monophasic and biphasic defibrillation. There was no statistical difference in rates of success between monophasic and biphasic cardioversion. For monophasic defibrillation, cardioversion was successful in 83.7 and 100% of patients with AF or atrial flutter, respectively, in a sample of 475 cardioversions performed. Two patients suffered a prolonged period of bradycardia associated with hypotension. Moore et al. (2014) [31], by analysing 974 elective cardioversions, obtained a sinus rhythm in 89.6% of cases. Four bradycardia, five transient heart blocks and two asystoles requiring cardiopulmonary resuscitation, and one stroke were the onset complications. Norton et al. (2016) [27] show an overall success rate of 93.4%. No major complications were reported. House et al. (2016) [33] discovered a success rate of 92% with rare adverse events that were generally bradycardia-related. Strzelczyk et al. (2017) [32] evidence that cardioversion for AF can be performed safely, with a 95% success rate by an APP. There were four adverse events: one transient ischaemic attack and three bradycardia requiring medication intervention. Purkayastha et al. (2023) [28] report a success rate of 87.68% on 341 patients who underwent DCCV, with no direct complication requiring readmission observed. Zaher et al. (2023) [34] obtained the restoring of sinus rhythm in 705 cardioversion (89.5%). One patient experienced ischaemic stroke within 24 h, despite adequate anticoagulation. There were three cases of severe bradycardia and three of transient respiratory depression.

**Figure 3:**
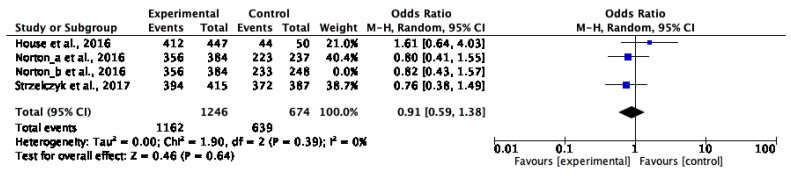
Meta-analysis showing the association between nurse-led DCCV and clinician control group efficacy and safety considering Norton et al. (2016) (a) data [27,32,33].

**Figure 4:**
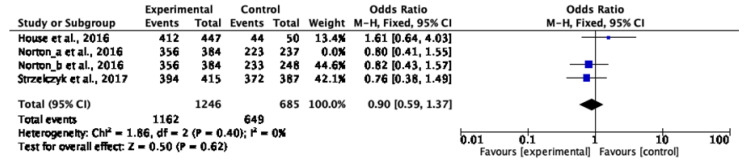
Meta-analysis showing the association between nurse-led DCCV and a clinician control group efficacy and safety considering Norton et al. (2016) (b) data [27,32,33].

We decided to synthesise the little data available using a meta-analysis methodology. Norton et al. (2016) [27] shared results with two different control groups. The first control group that we defined, Norton et al. (2016) (a) [27], compared nurse-led and medical DCCV. Norton et al. (2016) (b) [27] evaluated nurse-led and medical combined with nurse DCCV. Two different meta-analyses distinguishing the two analyses of the Norton et al. (2016) [27] study were conducted. Through the first meta-analysis (Figure 3) of the three studies evaluating the DCCV efficacy by experimental nurse-led intervention versus a control group, a statistically significant difference (OR 0.89; CI [0.58, 1.36]) of efficacy was not evidenced (Z = 0.54; *p* = 0.59). Similarly, the second meta-analysis (Figure 4) did not evidence a statistically significant difference (OR 0.90; CI [0.59, 1.37]) with a *p*-value of 0.62 (Z = 0.50). In both cases, the odds ratio value favoured nurse-led DCCV even if it was not statistically significant. It is possible to state that the result and correlated effect is not statistically inferior to that of other health professionals involved. Norton et al. (2016) [27] presented two different control groups’ data that were divided and analysed separately in the meta-analysis.

Norton et al. (2016) (a), Norton et al. (2016) (b) [27] and Strzelczyk et al. (2017) [32] reported that an intervention group, with autonomous nurse involvement, is more beneficial than control groups. House et al., 2016 [33], showed a control group preference (OR 1.61; CI [0.64, 4.03]). This datum is associated with a control group dimension that is significantly lower than the experimental group. No heterogeneity was found. Indeed, the X2 value was inferior to freedom degree, the X2 *p*-value was more than 0.10, and Higgins heterogeneity index in both analyses (0%) was less than 30%.

Furthermore, due to the limited number of studies, funnel plot elaboration, sensitivity analysis, and a subgroup analysis are not indicated.

The certainty of efficacy and safety in restoring sinus rhythm considering the grade approach and the Robins I evaluation of the three studies included in the meta-analysis is low due to the low prevalence of inefficacy between studies combined with the risk of bias addressed (Table 2).

### 3.4. Arrhythmia Type

The cardioversion service implemented consists of synchronised cardioversion of patients with stable clinical conditions. The atrial arrhythmia was mostly atrial fibrillation, but all studies also included patients with atrial flutter or atrial tachycardia. The prevalence of these arrhythmias was lower than that of atrial fibrillation. However, Moore et al. (2014) [31], Purkayastha et al. (2023) [28] and Shelton et al. (2006) [29] evidence that the success of atrial flutter DCCV in restoring sinus rhythm was more elevated.

### 3.5. Intervention Type and Medical Presence

The intervention studies delivered consist of a nurse-led direct current external cardioversion. To implement the cardioversion, a synchronised defibrillator function was utilised. Quinn (1998) [35] and Boodhoo et al. (2004) [30] provided monophasic defibrillator shock for cardioversion. Shelton et al. (2006) [29] analysed monophasic and biphasic shock cardioversion with no statistical difference in rates of success. House et al. (2016) [33], Moore et al. (2014) [31] and Zaher et al. (2023) [34] specify the use of a biphasic defibrillator to deliver the cardioversion. A nurse did not always deliver the cardioversion without the physical presence of a doctor in the same room, even if the cardioversion was nurse-led. Studies in which a nurse was alone and the medical cardiologist or anaesthetist staff was available in case of necessity but not physically present certainly include Boodhoo et al. 2004 [30] and Zaher et al. (2023) [34], and possibly include Moore et al. (2014) [31] and Purkayastha et al. (2023) [28].

### 3.6. Anaesthetic Support

The anaesthetic support delivered differs across studies (Table 1). Zaher et al. (2023) [34] clarify that the anaesthetic support is delivered by a nurse using an Etomidate bolus of 0.1 mg/kg, using other sedatives with cardiology support only if Etomidate was not successful. In the study of Boodhoo et al. (2004) [30], sedation was administered by a nurse, and cardiology was immediately available if required. Sedation characteristics are not specified. Purkayastha et al. (2023) [28] do not explain this aspect clearly. They report “access to medical and anaesthetic support if required”. All the other studies foresee the presence of anaesthesiology service. General anaesthesia is used in the study of Quinn, (1998) [35] and Shelton et al. (2006) [29]. Deep sedation with anaesthetic support is delivered in three studies: House et al. (2016) [33], Norton et al. (2016) [27] and Strzelczyk et al. (2017) [32]. Moore et al. (2014) [31] talk about brief anaesthesia.

### 3.7. Nurse Training

To autonomously deliver a DCCV for atrial arrhythmia, all studies included show that a nurse is required to have an advanced life support certificate. Boodhoo et al. (2004) [30], House et al. (2016) [33], Norton et al. (2016) [27], Quinn (1998) [35], Shelton et al. (2006) [29], Strzelczyk et al. (2017) [32] and Zaher et al. (2023) [34] also required a training or a period of supervision.

### 3.8. Secondary Outcomes

Boodhoo et al. (2004) [30] evaluated patient procedure tolerability, affirming that there was a high degree of patient satisfaction and willingness to repeat the procedure if indicate (90% of patients). Strzelczyk et al. (2017) [32] did not evidence a statistically difference between the satisfaction of patients who underwent the procedure before and after the advanced practice provider involvement. Boodhoo et al. (2004) [30] also studied cost-effectiveness, which correlated with waiting list. They reported an increased number of procedures, which translated into shorter waiting times. Norton et al. (2016) [27] observed a significant reduction in the length of stay for procedures conducted by nurses.

## 4. Discussion

Rhythm control in patients with atrial arrhythmia is indicated to restore the patient’s rhythm to normal. Rhythm control includes pharmacological cardioversion, external cardioversion, and catheter ablation [7,36]. It is necessary to evaluate the use of external cardioversion in patients with atrial fibrillation that does not terminate spontaneously [8]. As the studies included in the review show, the success rate for external cardioversion is high. Except for the results of Quinn (1998) [35]. Sinus rhythm is achieved in more than 80% of cases for all studies. Obtaining a sinus rhythm restoration and implementing an early rhythm control is important in reducing the risk of atrial remodelling [37] with correlated symptoms and death from cardiovascular causes, as well as improving quality of life [38].

DCCV is widely used in different clinical settings including emergency rooms, inpatient, or outpatient [39]. The studies analysed reflect this condition. DCCV has to be performed in a safe setting in which the patient is adequately monitored. This condition is in fact common to all studies included. Both inpatients and outpatients can receive DCCV. Indeed, the studies of Moore et al. (2014) [31] and Strzelczyk et al. (2017) [32] run cardioversion in inpatient and outpatient settings, while House et al. (2016) [33] and Norton et al. (2016) [27] consider an outpatient care unit.

Nurses may have an important role in the management of atrial arrhythmia. Rush et al. (2019) [40], with their systematic review, show that nurse-led atrial fibrillation clinics improve healthcare and quality of care outcomes. The services offered may include patient education, information-sharing, medication use and management, cardioversion service, and access to multiple treatment options.

Two studies [30,34] clearly implement the nurse-led DCCV intervention autonomously without the presence of cardiology staff. Only the study of Boodhoo et al. (2004) [30] completely implements this, including the anaesthetic aspect, by a nurse without the presence of an anaesthesiologist. Also, Zaher et al. (2023) [34] administer the sedation intervention with a predetermined etomidate protocol by a nurse, but they ensure the presence of a cardiologist back-up. Current ESC guidelines affirm that external cardioversion can be performed safely in sedated patients treated with intravenous i.v. midazolam and/or propofol or etomidate [4]. Deep sedation with these drugs is performed by House et al. (2016) [33], Norton et al. (2016) [27] and Zaher et al. (2023) [34]. Only the two studies by Quinn (1998) [35] and Shelton et al. (2006) [29] implement cardioversion with general anaesthesia.

Another aspect for which it is important to reflect on the nurse-led DCCV is nurse training. All studies state that the nurse involved must have specific training with advanced competences. Advanced life support training is a certification that is always requested. Boodhoo et al. (2004) [30], House et al. (2016) [33], Quinn (1998) [35], Shelton et al. (2006) [29], Strzelczyk et al. (2017) [32] and Zaher et al. (2023) [34] also add a variable degree of experience or supervision. The prevalence of advanced life support training was 100% among studies analysed. Each study could also request experience periods and/or a minimum number of cardioversion supervisions, but advanced training was always present. This result supported our decision to retain this grade of evidence in the summary of findings as moderate even if the results derived from observational studies.

However, it is clear that the sedation aspect, medical presence, and setting of development differ across the studies analysed. It could be interesting to reflect on the development of a specific nurse-led DCCV protocol, including guidelines and evidence from a systematic review, to improve safety implementation. A specific protocol with adequate training starting from advanced life support education can help implement nurse-led DCCV to obtain positive outcomes, which the studies demonstrated. Darrat et al. (2023) [41] recently demonstrated that a clearly defined and stepwise protocol can increase the success rates in restoring sinus rhythm in >99% of patients.

Considering only studies with two arms, we developed the first meta-analysis to synthesise the few data available on the topic that do not evidence a statistically significant difference in nurse-led DCCV efficacy (experimental group) compared to medical involvement (control group). The studies included were not randomised controlled studies, and the control group dimension was not always similar. This piece of information should guide future research to enrich this meta-analysis and confirm or redefine the relationship analysed. The relationship has to be interpreted also considering other care aspects. The absence of difference in the efficacy of DCCV led by a nurse or a clinician might, anyway, be a piece of information in support of the implementation of the intervention. If there is no risk difference, the nurse could be a healthcare professional able to independently implement the intervention, reducing clinician workload. This possible implementation should lead to other aspects of care considerations such as cost-effectiveness and possible waiting time reductions.

The quality of evidence with the grade approach for the efficacy and safety outcome was low because the studies included were not randomised controlled trials. Heterogeneity between studies was not evidenced. However, this result should be analysed also considering others studies not included in the meta-analysis. As appears with the Robins-I critical appraisal, the studies included in the systematic review had some limitations. However, except the study of Quinn (1998) [35], which had the smallest sample size, and even with publication dates ranging from 2004 with the study of Boodhoo et al. [30] to 2023 with the Zaher [34] study, all evidenced positive results and a nurse-led DCCV efficacy at least superior to 80%. This result has to be utilised with caution, and randomised controlled trials on nurse-led atrial fibrillation DCCV should be conducted. To create support for upgrading or downgrading the level of evidence discovered, future studies should start from an intervention delivered by the nurse without medical presence, with nurse-administered anaesthetic drugs, starting from advanced life support nurse training.

It is, however, important to underline that the interventions of studies included in the meta-analysis had some small differences that prevented generalizability. Future studies must consider aligning to the same intervention modality. The studies included considered DCCV in non-emergent situations. This is an important aspect to take into account for generalizability only in specific settings, but it may also represent a starting point for others considering different settings with appropriate evaluation.

The analysis of results must be guided by the awareness that this systematic review has some limits. It is important to consider that there is some heterogeneity in the settings and interventions in the studies included. This makes generalisation, synthesis, and meta-analysis interpretation difficult. Furthermore, the risk of bias in the study evaluation is not low. No study was, however, excluded due to the limited number of studies available and because a meta-analysis on the topic had not already been conducted.

Forest plot analysis showed an asymmetric distribution with a more important study concentration on the one hand that may be a potential publication bias. Potential studies with negative results may not be published. However, the limited number of studies means that stratified analysis and funnel plot elaboration are not recommended.

The development of other studies on the topic will be useful to add information and allow the synthesis of results for future implementation. As correctly evidenced by Boodhoo et al. (2004) [30], a nurse’s medicolegal considerations for each specific setting will be evaluated.

## 5. Conclusions

In our systematic review, we found that nurse-led DCCV is an important topic that appears safe, effective, and correlated with positive patient outcomes. Other evidence is necessary to clarify aspects of sedation and medical presence in support of the results of the studies that implement a completely autonomous nurse-led DCCV. Nurse-led DCCV improvement should be firstly guided by a clear definition and protocol of sedation. The nurse’s role and task must be declared in order to understand specific clinician or other health professional involvement. Nurses’ advanced life support training may be considered a first shared point of implementation in clinical practice. For safe implementation, it may be useful to evaluate shared international modalities of nurse-led DCCV in outpatient and inpatient settings.

## Figures and Tables

**Table 1:** Nurse training and anaesthetic aspects during DCCV.

	Study Design	Aim	Outcomes Measured	DCCV Nurse Training	Sedation Aspect and Medical Presence	Medical Presence in the Cardioversion Room (Cardiologist or Anaesthetist)
Quinn, 1998 [35]	Observational study (United Kingdom)	To perform an external elective direct current cardioversion by, or under the direct supervision of, the cardiac nurse specialist.	Efficacy Safety	Advanced life support training and minimum eight procedures under supervision.	General anaesthesia.	Present
Boodhoo et al., 2004 [30]	Prospective longitudinal study (United Kingdom)	To prospectively determine the short- and medium-term safety and effectiveness of a nurse-led cardioversion service.	Efficacy Safety Tolerability Cost effectiveness Waiting time	At least one year of coronary care unit experience, advanced life support certification, course of safe sedation practice, performed a minimum of 20 supervised sedation–cardioversion procedures.	Sedation administered by nurse.	Absent
Shelton et al., 2006 [29]	Retrospective study (United Kingdom)	To evaluate if nurse-led external direct current cardioversion is an effective method of restoring sinus rhythm in patients with persistent atrial arrhythmias.	Efficacy Safety Difference between monophasic or biphasic defibrillation	Senior nurses with advanced life support certification and a period of physician supervised cardioversion.	General anaesthesia.	Present
Moore et al., 2014 [31]	Prospective cohort study (Australia)	To evaluate the results and outcomes of a nurse led day case external direct current cardioversion.	Efficacy Safety	Up-to-date advanced life support training.	“Brief anaesthesia” not specified by whom it was administered.	Unclear
House et al., 2016 [33]	Observational retrospective study (Minnesota)	To analyse the outcome of patients whose elective DCCV was performed by an advanced practice provider.	Efficacy Safety	Be a certified nurse practitioner, have at least 1 year working experience in clinical cardiology and 6 months’ working experience in cardiac electrophysiology, with proficiency in arrhythmia interpretation, have performed a minimum of 25 elective cardioversions supervised directly by a cardiologist, current certification in advanced cardiac life support, demonstrate thorough understanding of the recent guidelines.	Deep sedation with methohexital sodium, etomidate or diprivan administered by anaesthesiology.	Present
Norton et al., 2016 [27]	Retrospective descriptive study (California)	To compare safety and effectiveness of a direct current cardioversion service performed by a medical doctor, a nurse practitioner under supervision or nurse practitioners.	Efficacy Safety Length of stay	National board certification as an acute care nurse practitioner and a certified cardiac device specialist, over 5 years of experience in electrophysiology and current basic life support and advanced cardiac life support certification.	Sedation with propofol administered by cardiac anaesthesiologist.	Present
Strzelczyk et al., 2017 [32]	Prospective study (Not clear probably Chicago, USA)	To prospectively determine the feasibility, safety, and efficacy of elective electrical cardioversion for atrial fibrillation when performed autonomously by a trained advanced practice provider using a guideline-directed protocol.	Efficacy Safety Patient satisfaction	Advanced cardiac life support certification and training by a cardiac electrophysiologist.	Deep sedation by an attending anaesthesiologist often with a nurse anaesthetist.	Present
Purkayastha et al., 2023 [28]	Retrospective cohort study (Chelmsford, England)	To establish whether there is a longer-term benefit and whether there are any late complications related to nurse-led direct current cardioversion service.	Efficacy Safety	Advanced life support certification.	Not clear.	Unclear
Zaher et al., 2023 [34]	Retrospective cohort study (Belgium)	To assess the safety and efficacy of a nurse-led elective direct current cardioversion using etomidate as sedation.	Efficacy Safety Sedation efficacy	Advanced life support certification and intensive care experience.	Sedation administered by nurse with etomidate.	Absent

**Table 2:** Summary of findings.

Nurse-Led External Direct Current Cardioversion Efficacy and Safety				
Patients or Population: Anyone with Atrial Fibrillation Who Undergoes a Nurse-Led DCCV Settings: Monitored Inpatient or Outpatient Setting Intervention: Nurse-Led DCCV Comparison: Medical-Led DCCV				
Outcomes	Odds Ratio	Number of Cardioversions	Certainty of the Evidence (GRADE)	Comments
Efficacy and safety in restoring sinus rhythm	Meta-analysis Figure 2: OR 0.89; CI [0.58, 1.36] Meta-analysis Figure 3: OR 0.90; CI [0.59, 1.37]	1630 (three studies)	⊕⊕⊝⊝ **Low ^a^** Due to the risk of bias	Low prevalence of inefficacy between studies and low prevalence of major complications.
Outcomes	Prevalence	Numbers of cardioversion	Certainty of the evidence (GRADE)	Comments
Professional training: Advanced Life Support	100%	4894 (nine studies)	⊕⊕⊕⊝ **Moderate ^b^** Due to imprecision	All studies foresee at least advanced life support nurse training.

^a^ Due to the overall risk of bias from moderate to critical of studies included. ^b^ Due to the lack of a clear consensus of the type of intervention delivered. ⊕⊕⊕⊕ High certainty; ⊕⊕⊕⊝ Moderate certainty; ⊕⊕⊝⊝ Low certainty; ⊕⊝⊝⊝ Very low certainty

## Data Availability

The original contributions presented in this study are included in the article. Further inquiries can be directed to the corresponding author.

## Supplementary Materials

The following supporting information can be downloaded at: https://www.mdpi.com/article/10.3390/nursrep15020032/s1, Table S1: Specific search strategies for each database; Table S2: ROBINS I assessment for DCCV safety and effectiveness; Information S1: Explanation of data used in the meta-analysis.

## Guidelines and Standards Statement

This manuscript was drafted against the preferred reporting items for systematic review and meta-analysis (PRISMA) statement criteria.
