# Supplementary material for: Nurse-Led Electrical External Cardioversion of Patients with Atrial Arrhythmia: A Systematic Review Update and Meta-Analysis
Source: Nurs Rep. 2025 Jan 23;15(2):32. doi: 10.3390/nursrep15020032 (PMC11858258; doi:10.3390/nursrep15020032)
Supplement: Supplementary file 1 [file nursrep-15-00032-s001.zip › nursrep-3388299-supplementary.pdf]

Nurse-Led Electrical External Cardioversion of Patients with Atrial Arrhythmia: A Systematic Review Update and Meta-Analysis

Dalia Caleffi[1], Luca Pingani[2], Sergio Rovesti[2], Domenico Cannizzaro[1], Paola Ferri[2]

[1]Clinical and Experimental Medicine PhD Program, Department of Biomedical, Metabolic and Neural Sciences, University of Modena and Reggio Emilia, Modena, Italy

[2]Department of Biomedical Metabolic and Neural Sciences, University of Modena and Reggio Emilia, Modena, Italy

Orcid

Dalia Caleffi: 0009-0009-8696-5447

Paola Ferri: 0000-0001-7761-7226

Sergio Rovesti: 0000-0002-1499-3897

Luca Pingani: 0000-0003-3428-8308

Domenico Cannizzaro: 0009-0001-6675-2788

Correspondence:
Full first name Caleffi, Dalia
Clinical and Experimental Medicine PhD Program, Department of Biomedical, Metabolic and Neural Sciences, University of Modena and Reggio Emilia, Modena, Italy.
via G. Campi 287, 41125, Modena, Italy

Supplementary Table S1: Specific search strategies for each database

Database	Search Strategy
**Cinahl and Psycinfo**	(atrial fibrillation OR auricular fibrillation OR atrial arrhythmias) AND (cardioversion OR electric countershock OR electric defibrillation OR electrical cardioversion OR electroversion OR external cardioversion OR DC cardioversion) AND (advanced practice nursing OR cardiovascular nursing OR critical care nursing OR cardiac nursing OR coronary care nursing OR nurse-led OR nurse-run OR nurse's role)
**Embase**	('atrial fibrillation'/exp OR 'atrial fibrillation' OR 'heart atrium arrhythmia'/exp OR 'heart atrium arrhythmia') AND ('cardioversion'/exp OR cardioversion OR 'defibrillator'/exp OR defibrillator OR 'electric shock'/exp OR 'electric shock') AND ('nurse'/exp OR nurse OR 'nurse led intervention'/exp OR 'nurse led intervention')
**Web of Science**	(atrial fibrillation OR auricular fibrillation OR atrial arrhythmias) AND (cardioversion OR electric countershock OR electric defibrillation OR electrical cardioversion OR external cardioversion OR DC cardioversion) AND (advanced practice nursing OR cardiovascular nursing OR critical care nursing OR cardiac nursing OR coronary care nursing OR nurse-led OR nurse-run OR nurse's role)
**Cochrane Library**	(atrial fibrillation OR auricular fibrillation OR atrial arrhythmias) AND (cardioversion OR electric countershock OR electric defibrillation OR electrical cardioversion OR electroversion OR external cardioversion OR DC cardioversion) AND (advanced practice nursing OR cardiovascular nursing OR critical care nursing OR cardiac nursing OR coronary care nursing OR nurse-led OR nurse-run OR nurse's role)
**Scopus**	(atrial fibrillation OR atrial arrhythmias) AND (cardioversion OR electroshock OR defibrillator) AND (nurse)
**PubMed**	(atrial fibrillation OR atrial fibrillation[mesh] OR auricular fibrillation OR atrial arrhythmias) AND (cardioversion OR electric countershock[mesh] OR electric defibrillation OR electrical cardioversion OR electroversion OR external cardioversion OR DC cardioversion) AND (advanced practice nursing[mesh] OR cardiovascular nursing[mesh] OR critical care nursing OR cardiac nursing OR coronary care nursing OR nurse-led OR nurse-run OR nurse's role)

Supplementary Table S2: ROBINS I assessment for DCCV safety and effectiveness.

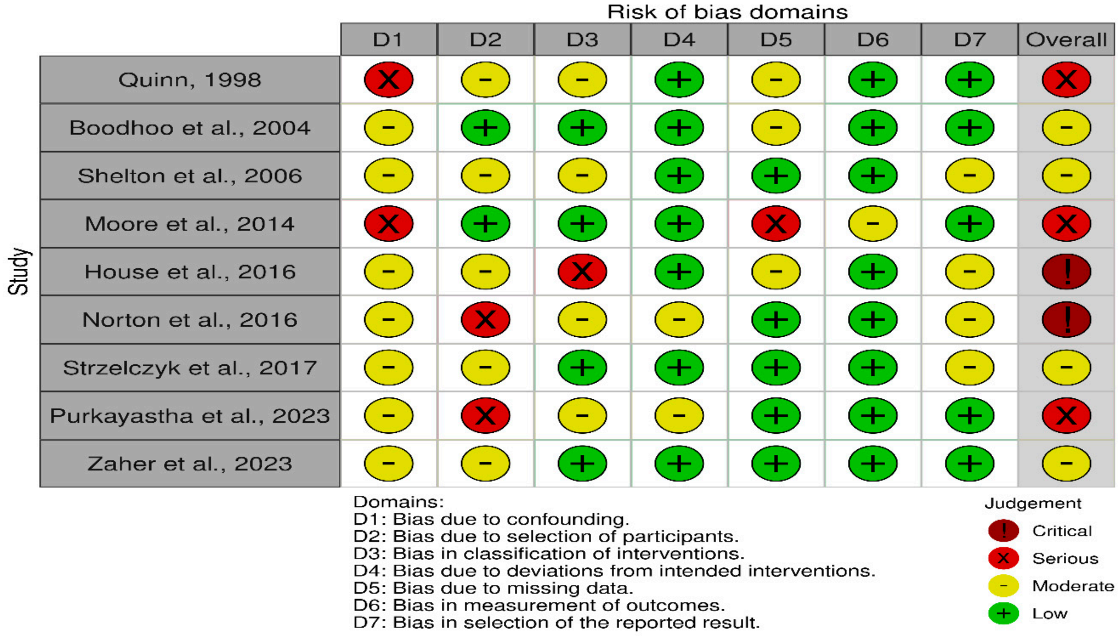

Supplementary Information S1: Explanation about data used in the meta-analysis

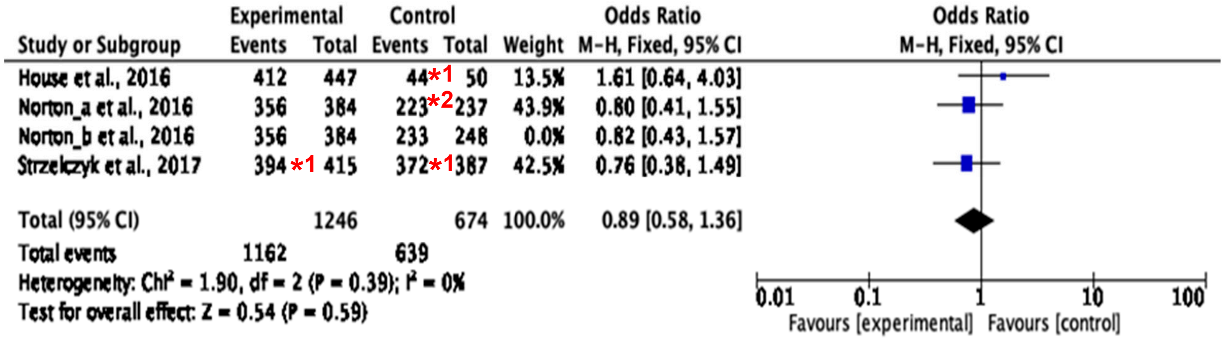

Study or Subgroup	Experimental Events	Experimental Total	Control Events	Control Total	Weight	Odds Ratio M-H, Fixed, 95% CI
House et al., 2016	412	447	44*1	50	13.5%	1.61 [0.64, 4.03]
Norton_a et al., 2016	356	384	223*2	237	43.9%	0.80 [0.41, 1.55]
Norton_b et al., 2016	356	384	233	248	0.0%	0.82 [0.43, 1.57]
Strzelczyk et al., 2017	394 *1	415	372*1	387	42.5%	0.76 [0.38, 1.49]
Total (95% CI)		1246		674	100.0%	0.89 [0.58, 1.36]
Total events	1162		639			

Heterogeneity: $Chi^2 = 1.90$, df = 2 (P = 0.39); $I^2 = 0\%$

Test for overall effect: Z = 0.54 (P = 0.59)

Study or Subgroup	Experimental Events	Experimental Total	Control Events	Control Total	Weight	Odds Ratio M-H, Fixed, 95% CI
House et al., 2016	412	447	44 *1	50	13.4%	1.61 [0.64, 4.03]
Norton_a et al., 2016	356	384	223 *2	237	0.0%	0.80 [0.41, 1.55]
Norton_b et al., 2016	356	384	233	248	44.6%	0.82 [0.43, 1.57]
Strzelczyk et al., 2017	394 *1	415	372 *1	387	42.1%	0.76 [0.38, 1.49]
Total (95% CI)		1246		685	100.0%	0.90 [0.59, 1.37]
Total events	1162		649			

Heterogeneity: Chi² = 1.86, df = 2 (P = 0.40); I² = 0%

Test for overall effect: Z = 0.50 (P = 0.62)

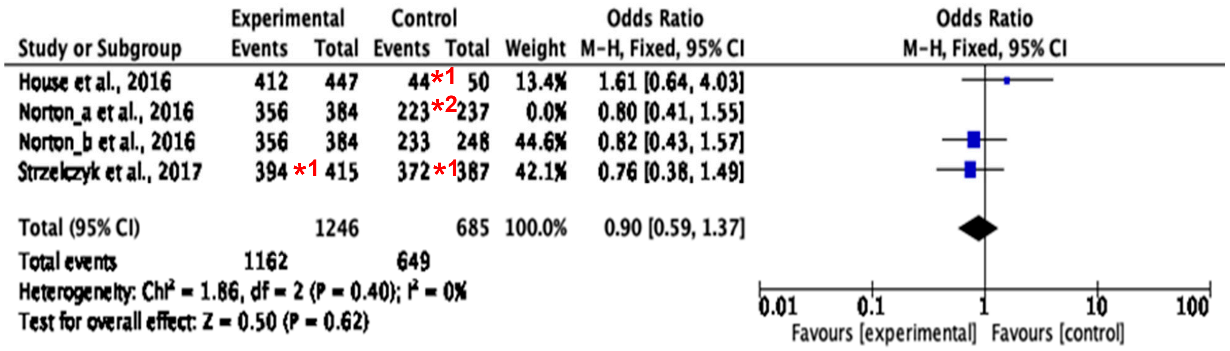

*1 Meta-analysis was conducted extracting data of events clearly declared by authors in the articles or mathematically derived from success frequency percentages.

*2 In table 2, item 2 (MD/NP) of Norton et al. (2016) we noticed a misprint. We contacted the corresponding authors to have confirmation, but we didn’t receive any response. The meta-analysis with the agreement of all authors was conducted with the value mathematically derived from the table.
